# Sibling sex, but not androgens, shapes phenotypes in perinatal common marmosets (*Callithrix jacchus*)

**DOI:** 10.1038/s41598-018-37723-z

**Published:** 2019-01-31

**Authors:** Brett M. Frye, Lisa G. Rapaport, Talia Melber, Michael W. Sears, Suzette D. Tardif

**Affiliations:** 1 Department of Biological Sciences, Clemson University, Clemson, South Carolina 29634 USA; 2 Department of Anthropology, University of Illinois, Urbana-Champaign, Urbana, Illinois 61801 USA; 3 Southwest National Primate Research Center, San Antonio, Texas 78227 USA

## Abstract

When offspring share a womb, interactions among fetuses can impart lasting impressions on phenotypic outcomes. Such intrauterine interactions often are mediated by sex steroids (estrogens and androgens) produced by the developing fetuses. In many mammals, intrauterine interactions between brothers and sisters lead to masculinization of females, which can induce fitness consequences. Many litter-bearing primates, though, seem to escape androgen-mediated litter effects, begging why? Here, we investigated how the sex composition (i.e., same- or mixed-sex) of litters influences perinatal outcomes in the common marmoset monkey (*Callithrix jacchus*), using a combination of physiological, morphological, and behavioural assays. We hypothesized that androgens from male fetuses would mediate developmental differences across litter types. We found that newborns (24–36 hours old) from same- and mixed-sex litters were indistinguishable by urinary androgen profiles, birth weights, morphometrics, and behaviour. However, monkeys born into same- and mixed-sex litters exhibited subtle morphological and neurobehavioral differences later in the perinatal period, independent of their androgen profiles. Our findings suggest that while androgens from male fetuses likely do not organize their siblings’ phenotypes, perinatal stimuli may initiate divergent developmental trajectories among siblings, which, in turn, promotes inter-individual variability within families.

## Introduction

The hormonal milieu of intrauterine environments can canalize behavioural^1^, anatomical^2^, health^3^, and reproductive outcomes^4^, which, in turn, can impart lasting fitness consequences^5,6^. While maternal hormones no doubt shape the ecology of the womb^7–9^, fetuses themselves are endocrinologically active^10,11^. Because a fundamental determinant of fetal endocrinology is sex^12^, intrauterine environments may vary with the sex of gestating fetuses. Thus, when offspring share a womb, the hormones that fetuses encounter – and consequent developmental trajectories – may well depend on whether they are born alongside brothers, or sisters, or both.

Although both male and female fetuses produce estrogenic precursors such as dehydroepiandrosterone (DHEA)^13^, males also produce gonadal steroids, i.e., androgens, that facilitate masculinization and defeminization^14–16^. These fetally-derived androgens can readily diffuse across amniotic membranes in some mammals (i.e., rodents)^17^, which can stimulate nearby fetuses to become masculinized^18^. These processes, in turn, can lead to anatomical, metabolic, behavioural, and even cognitive differences between males and females^19–21^. For example, vertebrate males typically outperform females in spatial orientation tasks^22–24^ because of androgens’ organizational effects on the neuroanatomy of the hippocampus^21^. Additionally, biomarkers of masculinization (e.g., 2D:4D digit ratios) may portend pathophysiological risks later in life^25^.

The consequences of inter-fetal transfer of androgens have been well documented in rodents, lagomorphs, and ungulates (e.g., *Marmota marmota*^26^; *Oryctolagus cuniculus* and *Marmota flaviventris*^27^; *Ovis aries*^28^; and *Sus scrofa*^29^). However, the biological salience of this phenomenon has received less attention in other mammalian taxa, such as non-human primates. The few investigations that have assessed the developmental sequelae of prenatal androgen exposure in litter-bearing primates have yielded mixed results. Litter effects were largely negligible in eight callitrichine and strepsirrhine species (i.e., *Leontopithecus rosalia*, *Saguinus oedipus*, *Varecia variegata*, *Varecia rubra*, *Microcebus murinis*, *Mirza coquereli*, *Cheirogaleus medius*, and *Galago moholi*^30,31^). Others (i.e., *Callithrix jacchus* and *Leontopithecus rosalia*), though, exhibited some evidence of sex-dependent fetal interactions^31,32^. A major limitation of these previous surveys is the absence of direct measurements of the mechanism – fetal androgens – proposed to mediate differences in behavioural, reproductive, and survival outcomes. Rather, researchers used litter sex ratios, i.e., whether an individual is born into same- or mixed-sex litters, as a proxy for androgen exposure. These studies also focused on the potential detriments of prenatal androgens on female monkeys (but see^30^). Assays of circulating androgens in both male and female neonates are therefore needed to clarify the mechanistic underpinnings of sex-specific litter effects.

We explored the links among fetal androgens, litter composition, and early developmental trajectories in common marmosets (*Callithrix jacchus*) at the Southwest National Primate Research Center (SNPRC) using a combination of longitudinal records and experimental data that was designed specifically for this project (Table 1 and see Methods).

**Table 1:** Samples sizes–partitioned into individuals born into same- and mixed-sex litters–for each portion of the project.

Metric	Females		Males	
	Same-sex Litters	Mixed-sex Litters	Same-sex Litters	Mixed-sex Litters
Urinary Androgens	12	8	4	6
Perinatal Weights	48	81	31	81
Morphometrics	24	39	13	46
2D:4D Ratio	16	21	13	22
Marmoset Assessment Test (MatScore)	24	38	13	43
Primate Postnatal Neurobehavioural Assessment Scale for Marmosets (PPNAS-M)	6	11	4	7

Our sample consisted of marmosets born to twin and triplet litters of known sex composition, and we compared monkeys born to mixed-sex (i.e., at least one full-term male and female) litters to monkeys from same-sex (i.e., all male or all female) litters. These small-bodied monkeys provide an excellent “natural experiment” in which to investigate this phenomenon because they routinely produce multizygotic litters^33,34^. As such, there is natural variation in the sex composition – and ostensibly prenatal androgens – across litters. We examined the urinary androgen concentrations of neonatal marmosets (24–36 hours following birth) using enzyme immunoassays (EIAs). We chose to use EIAs, instead of liquid chromatography or tandem mass spectrometry, because we were limited by the small volumes of urine (<20 µL) produced by the neonates. This choice restricted the specificity of androgen compounds that we could detect^35^. These small volumes also constrained our ability to control for variation in urinary excretion dynamics (i.e., via concentration creatinine or specific gravity). We therefore refer to our results as “androgen” concentrations (ng/ml urine) because the antibody in this assay is known to cross-react with androstenedione and dihydrotestosterone (other androgen metabolite cross-reactivity<2%)^35,36^ (see Methods).

Our main objectives were to determine whether males 1) actually exhibited higher androgen concentrations and 2) appreciably altered the hormonal milieu of the uterus such that siblings in a litter with males reflect high-androgen environments by displaying higher urinary androgens. Androgens are not the only mechanism through which the presence of a male in the prenatal environment might alter development in litter mates (e.g., anti-Müllerian hormone (AMH)^37^). Therefore, we explored possible developmental divergence in morphology and behaviour across litter compositions in a larger dataset for which litter sex ratio was known but prenatal androgen profiles were not.

We expected that if androgens from male fetuses were transferred to siblings *in utero*, then neonatal individuals in all-male or mixed-sex litters should exhibit higher androgen concentrations than individuals from all-female litters. Intriguingly, our investigation revealed that androgens from male fetuses likely do not instigate divergent developmental trajectories in common marmoset monkeys, given the finding that newborns (24–36 hours following birth) from different litter compositions were indistinguishable by their morphology, behaviour, and, most importantly, their urinary androgen profiles. Contrary to our findings on the day following birth, infants that were born into same- versus mixed-sex litters exhibited some subtle morphological and behavioural differences later in the perinatal period (i.e., at postnatal days 15 and 30). This pattern suggests that either the effects of intrauterine factors become evident as marmosets age, sex-dependent interactions during the perinatal period impact development, or a combination of pre- and postnatal factors together determine developmental outcomes in common marmosets.

## Results

### Urinary Androgens

**Figure 1:**
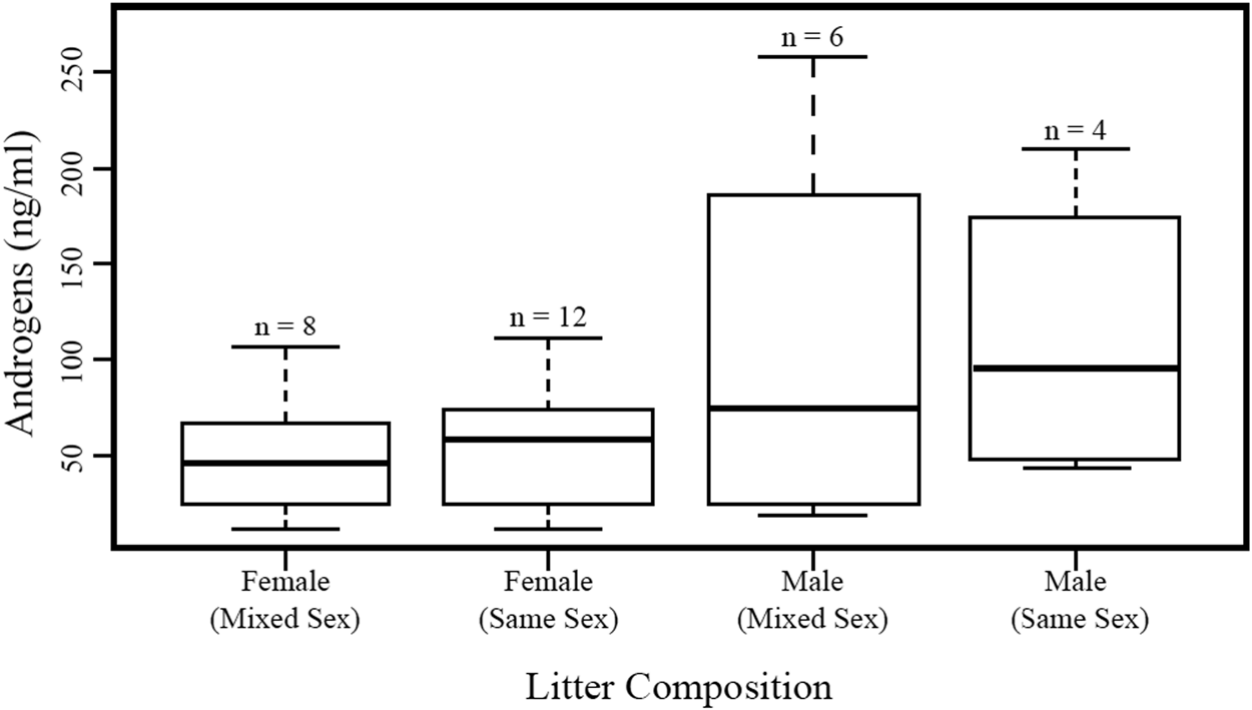
Urinary androgens (ng/ml urine) in neonatal marmosets born into same- and mixed-sex litters. Observed values showed no relationship between the sex composition of the litter and urinary androgen profiles.

**Figure 2:**
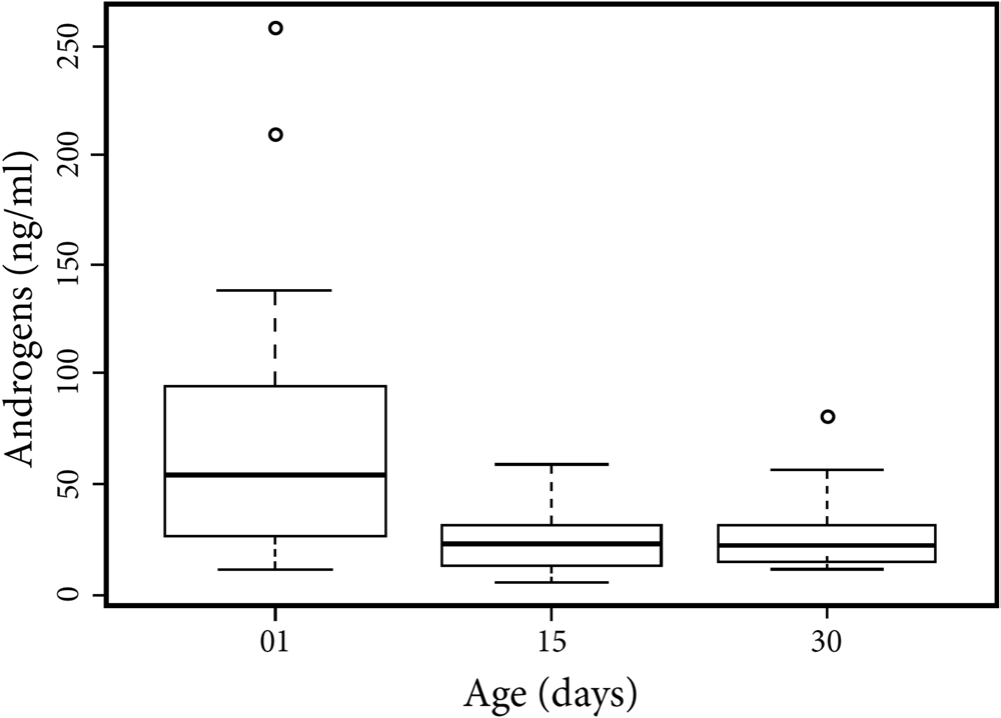
Androgens (ng/ml urine) in perinatal marmosets (postnatal days (PD) 01–30). Urinary androgens decreased over the perinatal period for all infants notwithstanding the sex composition of the birth litter.

We found no significant differences across litter type or sex in urinary androgens in neonatal marmosets (GLMM: t = −0.418; η^2^ = 0.0127; p = 0.675; Fig. 1). We did detect considerable variation among litters, though, with infants from the same litter exhibiting similar urinary androgen concentrations 24–36 hours following parturition (Supplementary Figure 1). Perinatal (i.e., postnatal days (PD) 15 and 30) marmosets from same- and mixed-sex litters did not differ in average urinary androgen concentration (GLMM: t = 0.570; η^2^ = 0.0170; p = 0.569). However, the concentrations of urinary androgens decreased over the perinatal period (GLMM: t = −2.559; η^2^ = 0.161; p = 0.011 Fig. 2).

Considering that we observed considerable variation among litters, we subsequently explored whether neonatal androgens, regardless of sex, predicted perinatal outcomes via linear mixed models. Urinary androgens were unrelated to birth weight (GLMM: F_1,22_ = 1.575; η^2^ = 0.131; p = 0.227) and body weight on postnatal day 30 (GLMM: F_1,10_ = 0.964; η^2^ = 0.268; p = 0.356). Further, there was no relationship between neonates’ urinary androgens and behaviour in either the MatScore (PD 01) or PPNAS (PD15 & PD30) procedures (CLMM: p > 0.05).

### Morphometrics

**Figure 3:**
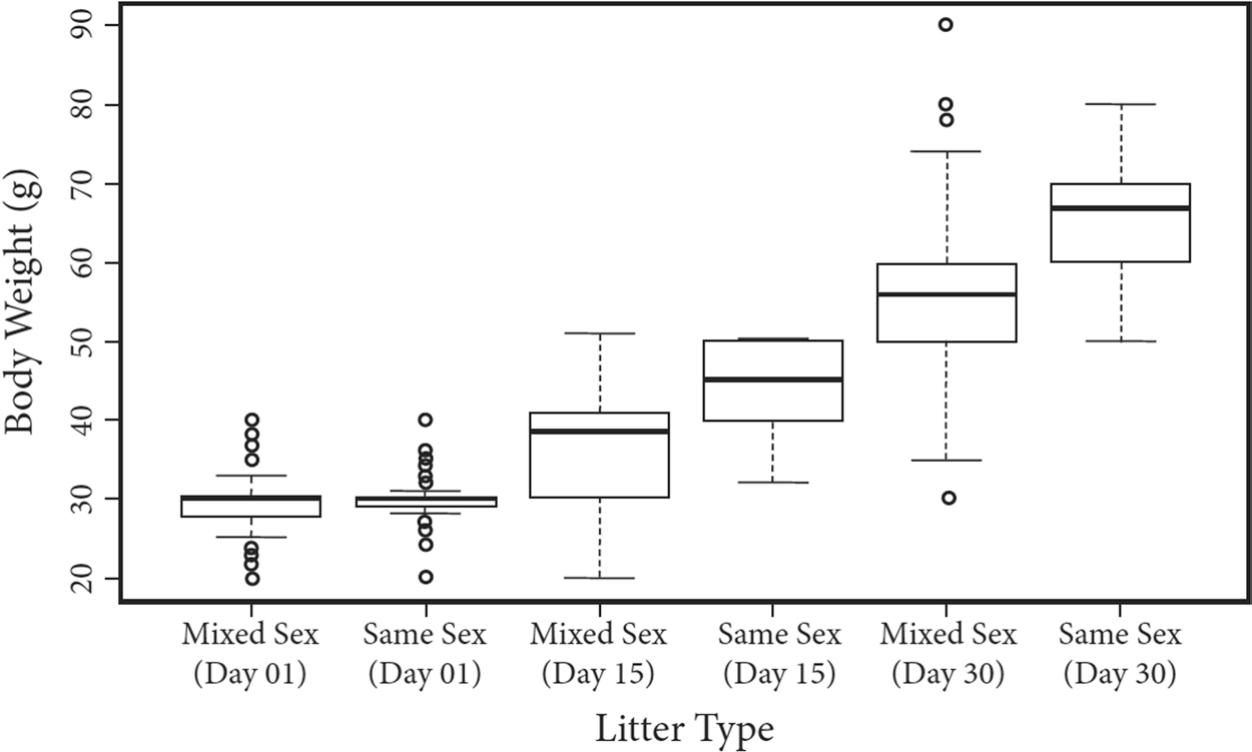
Body weights of perinatal marmosets (postnatal days (PD) 01–30) born into same- and mixed-sex litters. Both male and female marmosets from same-sex litters were heavier than individuals born into mixed-sex litters. This difference emerged later in the perinatal period (i.e., PD 15 & PD 30), but was not present at birth.

Marmoset infants, regardless of sex, from same-sex litters were larger than those born into mixed-sex litters, and these differences appeared later in the perinatal period (i.e., at PD15 and PD30) (GLMM: F = 10.855; η^2^ = 0.014; p < 0.001; Fig. 3). However, while we detected significant differences in overall body weight, our generalized linear mixed models did not reveal differences between same- and mixed-sex infants’ biparietal diameters, thigh-lengths, upper-arm lengths, abdominal circumferences, or crown-rump lengths (Supplementary Table 4).

Our preliminary analysis of animals’ digits revealed that the measurement method (i.e., photos or prints) differed significantly. As such, we conducted separate analyses for each dataset to investigate whether litter type predicted an individual’s 2D:4D ratio. We did not detect any differences in 2D:4D ratios while using prints of infants’ hands. We did, however, detect differences among different-aged marmosets using photos of individuals’ feet (t = 3.390; η^2^ = 0.087; p = 0.001). That is, older marmoset infants exhibited greater 2D:4D ratios on their toes than did younger infants.

### Infant Neurodevelopment and Behaviour - MatScore

We employed the Marmoset Assessment Test (“MatScore”) procedure to test for behavioural differences on infants’ first day of life^38^ (see Methods). After removing several behavioural variables that showed evidence of multicollinearity (i.e., clasping 2, righting 2, rooting (left side), and auditory orientation (left side) (for additional information about correlations, see Supplementary Table 1)), we conducted a series of cumulative link mixed models with litter type and sex as our predictor variables and litter as a random effect. We did not detect any significant behavioural differences between infants from same- and mixed-sex litters on the first day of life (Table 2). Additionally, overall MatScores did not depend on the sibling’s sex. However, our model did reveal sexual dimorphism in two behaviours. That is, males scored lower in tests of grasping (CLMM: z = −2.220; p = 0.026) and rooting (CLMM: z = −1.949; p = 0.050).

**Table 2:** Cumulative linked mixed models with fixed effects of litter type (same- versus mixed-sex) and sex on MatScore behaviours on neonatal marmosets (i.e., postnatal day 01). The sex composition of the litters did not impact behavioural performance on any of the assays in this procedure. Statistically significant differences are indicated in bold.

Response Variable	Fixed Effect	Estimate	SE	${\boldsymbol{z}}$	*P*
*Crawling*	Litter Type	−0.243	0.745	−0.326	0.744
	Sex	−0.836	0.700	−1.194	0.232
	Litter Type × Sex	−16.781	1186	−0.014	0.989
*Clasping*	Litter Type	−0.433	0.641	−0.675	0.500
	Sex	−0.410	0.536	−0.764	0.445
	Litter Type × Sex	−2.145	2.425	−0.885	0.376
*Righting 1*	Litter Type	0.082	0.810	0.102	0.919
	Sex	0.195	0.624	0.313	0.754
	Litter Type × Sex	14.379	838.9	0.017	0.986
*Grasping (1)*	Litter Type	1.200	0.679	1.766	0.077
	Sex	−1.224	0.551	−2.220	**0.026**
	Litter Type × Sex	12.701	419.4	0.030	0.976
*Grasping (2)	Litter Type	−0.075	1.162	−0.065	0.948
	Sex	−0.339	0.957	−0.354	0.723
	Litter Type × Sex	—	—	—	—
*Vertical Orientation	Litter Type	−0.022	0.856	−0.026	0.979
	Sex	0.499	0.787	0.635	0.526
	Litter Type × Sex	—	—	—	—
*Rooting	Litter Type	−0.716	0.533	−1.343	0.179
	Sex	−0.859	0.441	−1.949	**0.050**
	Litter Type × Sex	—	—	—	—
*Auditory Orientation	Litter Type	0.370	0.490	0.756	0.450
	Sex	−0.339	0.461	−0.736	0.461
	Litter Type × Sex	—	—	—	—
*Overall MatScore*	Litter Type	0.133	0.554	0.241	0.809
	Sex	−0.529	0.460	−1.150	0.249
	Litter Type × Sex	−1.735	1.775	−0.978	0.328

*Model is rank-deficient, so interaction cannot be determined.

### Infant Neurodevelopment and Behaviour – PPNAS-M

**Figure 4:**
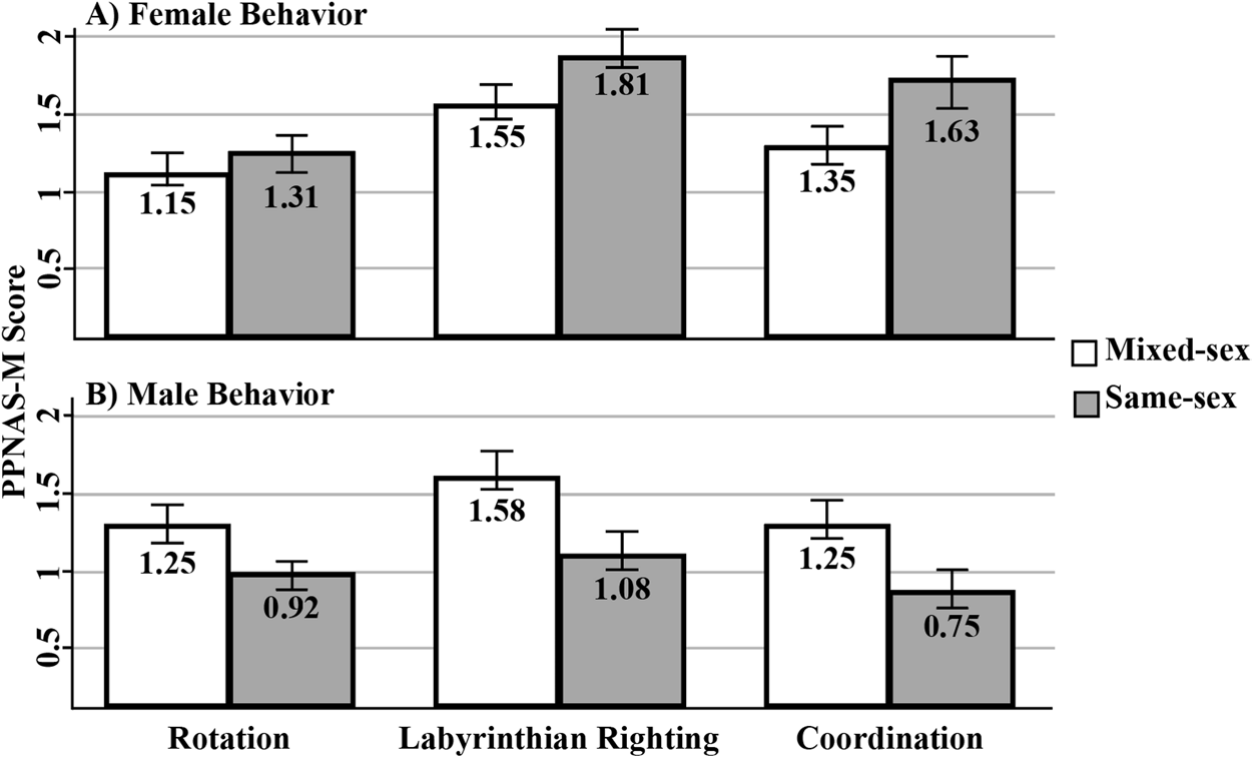
Effect of litter type (i.e., same- versus mixed-sex) on marmoset infants in three behavioural categories – rotation, labyrinthian righting, and overall coordination. Females that were born into mixed-sex litters scored lower than females born into same-sex litters (**A**), whereas males born into mixed-sex litters scored higher than males born into same-sex litters (**B**).

We used the Primate Postnatal Neurobehavioural Assessment Scale for Marmosets^39^ (“PPNAS-M”) to examine behavioural differences later in the perinatal period (i.e., PD15 & PD30) (see Methods for full procedure). Like the MatScore, many of the PPNAS-M tasks showed signals of multicollinearity (i.e., visual following, duration of looking, tactile response, range of, power during tests, rooting, auditory orientation, crawling, palmar response, response speed, head positioning while prone, and head positioning while supine (for additional information about correlations, see Supplementary Table 1)) and were omitted from subsequent analysis. In the remaining models, we detected a behavioural difference between males and females - aversion to being inverted - with males showing lower levels of distress to the testing protocol than did females (CLMM: z = −2.060; p = 0.039). Males and females also differed in their overall PPNAS-M scores. Males tended to exhibit lower PPNAS-M scores than females, however, this difference did not satisfy our predetermined criteria for statistical significance (CLMM: z = −1.917; p = 0.055). Marmosets born to same- and mixed-sex litters exhibited distinct behavioural profiles in the following assays: rotation, labyrinth response, and general coordination (Fig. 4). These differences in behaviour were sex-specific – while males born into same-sex litters exhibited lower behavioural scores than males born into mixed-sex litters, females born into same-sex litters exhibited higher behavioural scores than females born with brothers (CLMM: Rotation: z = −1.931, p = 0.053; Labyrinthian Righting: z = −2.223, p = 0.026; Overall Coordination: z = −2.276, p = 0.006).

## Discussion

Our investigation suggests that androgens from male fetuses do not instigate divergent developmental trajectories in common marmoset monkeys. Despite the lack of evidence for androgen-mediated effects, we did detect subtle differences among infants born into same- versus mixed-sex litters: male and female marmosets that were born into same-sex litters weighed more than marmosets born with opposite-sex siblings later in infancy (Fig. 3). We also detected behavioural differences in perinatal marmosets using the PPNAS-M protocol. That is, individuals born into same- and mixed-sex litters exhibited distinct behavioural profiles with regard to labyrinthian righting, rotational orientation, and general coordination (Fig. 4). These trends were sex-specific: females that were born with sisters (same-sex) exhibited greater capabilities than females born with brothers (mixed-sex), whereas the opposite was true for males. That is, males from same-sex litters exhibited lower scores that males that were born with sisters (Fig. 4). These results suggest that alternative mechanisms underlie the links between litter sex composition and developmental trajectories in common marmosets.

This is the first study, to our knowledge, in which researchers have directly measured the clearance of androgens from peripheral circulation through neonatal urine. Although longitudinal studies of individuals’ endocrine profiles before and after parturition do not exist, cross sectional experiments suggest that sexual dimorphism in androgen titers persist across the pre- and postnatal periods in rodents^11,40^. Further, testosterone has been shown to persist in callitrichine urine for at least two days following exposure^41^. We therefore assumed that urinary androgens 24–36 hours following parturition provided a reliable proxy for the hormonal environment individuals experienced *in utero* immediately prior to birth.

Our study parallels others’ attempts to discern the links between gestational androgens and offspring sex in callitrichine primates. French *et al*.^36^, demonstrated that marmoset (*C. kuhlii*) mothers exhibit a high degree of variation in their urinary androgen profiles during pregnancy. The sex composition of litters, though, did not affect maternal androgen profiles in their sample. Urinary androgens in pregnant marmosets, therefore, are probably maternally-derived. We, too, detected considerable variation in androgen profiles across pregnancies, independent of litter composition (Supplementary Figure 1). Given that urinary androgen concentrations at ~36 hours after birth likely reflect uptake from the intrauterine environment, whereas concentrations on postnatal days 15 and 30 reflect the infant’s physiology, our results suggest that maternal physiology is a principal factor determining the hormonal milieu fetuses encounter *in utero*. However, other obstetric factors (e.g., labor onset and duration, parity, placental physiology, and gestational age) also may have influenced fetal androgen profiles, as has been demonstrated in assays of umbilical cord blood in human neonates^42^. These additional factors may underlie our findings of changing 2D:4D ratios in marmoset infants (i.e., older marmosets exhibited greater 2D:4D ratios than younger marmosets). Future analyses which incorporate maternal androgens and such obstetric factors are needed to clarify the relationships between androgens and biomarkers of prenatal androgen exposure (e.g., 2D:4D ratios and ano-genital distance) in common marmosets and other primates.

Infants in our sample showed a marked decrease in androgens over the perinatal period (Fig. 2). Our study subjects also exhibited a high degree of variation in their androgen profiles during this period (Fig. 2). These findings are comparable to those described by Abbott and Hearn^43^, who also detected considerable variation in androgen profiles during male marmosets’ first 100 days of life (range: 0–13 ng/ml). In their sample, plasma testosterone peaked around postnatal day 40, whereas infants in our sample exhibited the highest urinary androgens on the first day of life. A key difference between these studies is that we quantified androgens on postnatal day 01 and included both males and females in our sample, whereas Abbott and Hearn collected data for androgen titers beginning on postnatal day 15 and measured androgens in males alone. Notwithstanding these differences, our data, along with others’ (e.g.,^1,36^) suggest that considerable variation in androgen production may be representative of marmoset monkeys. These findings add to the growing narrative that characterizes marmosets as exceedingly variable in physiological (e.g., neuroendocrine responses^44^), behavioural (e.g., foraging^45^, anxiety-like behaviour^46^, and boldness^47^), and reproductive (e.g., pregnancy hormones^36^; placental architecture^48^; and parental effort^49^) domains.

Another factor that may have contributed to the absence of sex-driven differences in urinary androgens is the action of the adrenal fetal zone (FZ). Both male and female neonates express an adrenocortical FZ at birth^50^, and this region is responsible for secreting androgenic precursors such as dehydroepiandrosterone (DHEA) and its sulfo-conjugate (DS)^51,52^. These compounds may have acted as precursors to the androgens (i.e., androstenedione) excreted in the marmoset infants’ urine, thereby obscuring any signal from gonadal action. We are currently unable to determine how the FZ impacts urinary androgen profiles in neonatal marmosets. The relationship between DHEA and androstenedione, though, is complex and depends on the action of several enzymes and cofactors (i.e., P450c17, HSD3B, POR, and CYTB5)^52^. For example, low levels of the enzyme 3ß-hydroxysteroid dehydrogenase (HSD3B) are required for DHEA production because HSD3B inhibits in the biosynthesis of DHEA. However, increases in cytochrome b5 (CYTB5) facilitates the production of DHEA^52^. Whether these dynamics vary with sibling sex remains a mystery. Teasing apart these relationships may reveal the mechanistic underpinnings of variation in infant development.

Although we did not detect any differences in androgen excretion across litter types, this finding does not exclude the possibility that marmoset fetuses may have evolved protective mechanisms which effectively shield individuals from hormonal products from their siblings. French *et al*.^31^ discovered that callitrichines have several nonsynonymous mutations in the coding regions for genes mediating sexual differentiation. These regions were different from primate species that typically produce singletons (i.e., humans, macaques, and squirrel monkeys). The products of these candidate genes are integral in processes associated with the metabolism, transport, and reception of anti-Müllerian hormone (AMH) and androgens. French *et al*.^31^ also discovered polymorphisms in callitrichine aromatase–an enzyme that converts androgens into estrogens–which may confer protections against gestational androgens. Conversely, Bradley *et al*.^30^ did not find evidence for duplication events in the gene coding for aromatase (CYP19A1) in two callitrichine species (i.e., *Callthrix jacchus* and *Saguinus oedipus*). Bradley *et al*.^30^ propose that placental aromatase, rather than gonadal aromatase, instead might act to shield female marmosets from sibling-derived androgens. Future research of these genetic and epigenetic factors are needed to clarify how genetic mechanisms might mitigate the effects of shared intrauterine environments.

The finding that offspring from same-sex litters were heavier than offspring born into mixed litters is surprising considering that male callitrichine growth rates do not differ from those of female conspecifics^53,54^. Nor do females from same- and mixed-sex common marmoset (F_1,1194_ = 0.05; P = 0.828) and golden lion tamarin (*Leontopithecus rosalia*) ((F_1,306_ = 1.45; P = 0.229) litters exhibit distinguishable growth rates from birth to maturity (unpublished data). Although we note that the effect size detected in our analysis was small (η^2^ = 0.014), acute differences early post-natal care could explain the differences we observed in the perinatal weight profiles. Over the first 3–4 weeks of life, marmoset infants are carried almost exclusively by their parents, with mothers carrying most often during the first two weeks following parturition^55,56^. Marmoset mothers are notoriously flexible in the quality of care that they provide^57,58^. Some of this variation is driven by differences in the timing of conception during the postpartum estrus – marmoset mothers that conceive shortly after parturition reduce parental care compared to mothers that conceive later in the postpartum period^57^. These differences may stem from trade-offs between the energy allocated for current versus future reproduction. That is, when conception occurs during the early postpartum period, mothers must concomitantly gestate, nurse, and carry their offspring, which is energetically demanding^59,60^. When conceptions are delayed until infants are older, the energetic burdens may be less acute. While we did not investigate the relationship between litter composition and the timing of subsequent conceptions, future examinations of these processes could illuminate potential relationships between litter sex composition, maternal condition, maternal investment, and developmental outcomes.

An alternative, although not mutually exclusive, explanation for differences in same- versus mixed-sex litters’ weights may reflect differences among parents’ levels of kin recognition for infants born into same- versus mixed-sex litters. While uncommon in most mammals, marmosets regularly exhibit genetic chimerism. That is, because of inter-mingling of fetal stem cells during prenatal development, marmosets often contain some of their siblings’ genetic material^61–63^. However, levels of chimerism vary across individuals. As such, marmosets may exhibit varying levels of relatedness to family members, which in turn could manifest as variation in the quality or amount of care infants receive. In the single study exploring relationships between infant chimerism and parental behaviour, Ross *et al*.^61^ discovered that marmoset mothers carry chimeric infants less than non-chimeric infants. Marmoset fathers exhibited the opposite trend. That is, fathers actually provided superior care to chimeric offspring. If marmosets that are born to mixed-sex litters exhibit higher levels of detectable chimerism than do infants born into same-sex litters, then differential care from parents during the early postnatal period could explain the differences in perinatal weights among infants born with same- or opposite-sex siblings.

Although sources of variation in parental care offers an interesting *a posteriori* explanation, differences in the competitive capabilities of the marmoset infants themselves could impact parental investment strategies, leading to differences in early body condition. Callitrichines are cooperative breeders in which a single, dominant pair typically monopolizes reproduction within the group^64^. Marmosets groups therefore exhibit considerable skews in reproductive success^65,66^. This system also ensures that a marmoset’s fiercest competitors are often family members^66,67^. Within families, competition within cohorts can start early in life, with “twin fights” beginning around 180 days postpartum. Such agonism between marmoset siblings born into same-sex litters is typically more frequent than in mixed-sex pairs^68^. If marmoset infants can expect to engage in agonistic interactions with siblings of the same-sex, adaptive strategies may have evolved which prompt these individuals to pursue developmental strategies that maximize growth and competitive abilities. On the other hand, infants born into mixed-sex litters might expect lower levels of agonism with their siblings. Adaptive developmental strategies for individuals may instead be typified by slower growth patterns during the early perinatal period.

We also detected behavioural differences in perinatal marmosets using the PPNAS-M protocol. Since we conducted this assay on postnatal days 15 and 30, we cannot exclude the possibility that differences existed on the days following parturition. We therefore propose that these differences might be attributed to gestational stimuli, postnatal stimuli, or an interaction of the two. The behaviours in which we detected divergence are associated with development of the visual-vestibular system^69^. Proper development in these systems is critically important for arboreal primate infants, which must orient to their carriers and objects in their environments. This system also is critical for balance and overall coordination when moving about in complex environments^69^. In naturalistic settings, differences in the development of the visual-vestibular systems may impact the timing of locomotor independence, which, in turn, could instigate developmental differences in dispersal and foraging capabilities^70^.

Selective pressures for the timing of development in these systems may vary across litter compositions. In marmosets, females procure reproductive opportunities by either inheriting a breeding position within natal territories or by emigrating to neighboring groups^71^. As such, competition among same-aged sisters may have selected for fast developmental schedules, with females attaining locomotor independence more quickly if they are born with sisters than if born with brothers. Selection for intra-sexual competition among brothers instead may be relaxed because males have increased opportunities for mating both in and outside of the group^72^. Therefore, males that are born with brothers might delay locomotor independence as infants in order to garner resources from group members. This trade-off might, in turn, enhance males’ body conditions upon maturation.

Alternatively, being born with brothers might generally disadvantage siblings, independent of androgens, thereby promoting slower development of the visual-vestibular systems in both sexes. Less-developed infants at postnatal days 15 and 30 could result from variation in gestation ages. That is, if pregnancies that include males are shorter in duration, marmosets may be born less developed than infants that gestated for longer periods of time. In humans, gestations with male offspring are typically shorter than gestations with daughters^73^. Further, in preterm human twins, female-female pairs exhibited the longest gestations, whereas male-female and male-male twins were born approximately two weeks sooner^74^. These differences have been attributed to differences in prenatal growth patterns between males and females. Although we did not measure the duration of gestations in this study, hormonal monitoring during the postpartum estrus could help to clarify whether gestation lengths vary with the sex composition of litters.

Our study differed from previous studies in that we investigated the differences between individuals born into same- versus mixed-sex litters rather than focusing on females. Because of this difference, others mostly omitted analyses exploring potential outcomes in isosexual male litters. However, in a subset of their analyses, Bradley *et al*.^30^ found that male cotton-top tamarins (*Saguinus oedipus*) that were born with male co-twins experienced higher mortality than males that were born with sisters (Fishers exact: *p* = 0.03), indicating that male-male competition may be an important selective force in callitrichines. Considering these and our results together, future analyses should aim to investigate the mechanisms underlying the links between litter sex composition and development, reproduction, and survival in both male and female callitrichines.

We have outlined several possibilities for the mechanisms that might underlie phenotypic variation stemming from the early ontogenetic environments. However, this list is not exclusive. For example, given that alloparental care is particularly important for rearing marmoset offspring^75,76^, and individuals have been shown to vary in the levels of care they provide^77^, future investigations should investigate whether non-parental care-givers provide different levels of care depending on the sex composition of litters. Investigations using other biological samples (e.g., feces, hair, or plasma) may also provide clues to the associations between hormones measured in excreta and those acting on target tissues. Lastly, the preponderance of evidence in this area of inquiry has been conducted on captive animals. As such, investigations of these phenomena in wild animals are key. Together, such avenues will advance our understanding how pre- and post-natal environments shape developmental outcomes.

## Methods

### Study Subjects

Our total sample consisted of 202 common marmoset infants (n_male_ = 95; n_female_ = 107) that were born to 116 different litters between May 2003 and September 2017 at the Southwest National Primate Research Center (SNPRC), San Antonio, TX. We combined longitudinal records from the SNPRC and experimental data that was designed specifically for this project. Thirty marmoset infants (n_male_ = 10; n_female_ = 20) were born from January 2016 – June 2016 at the SNPRC. For these animals, we collected urine, morphometric, digit lengths, and behavioural data (i.e., MatScore and PPNAS-M). Historical records of infants’ weights, morphometrics, digit ratios, and behaviour (i.e., MatScore) made up the remaining portion of the data (n_male_ = 85; n_female_ = 87). We combined the historical and experimental datasets for our analyses of body weights, 2D:4D ratios, morphometrics, and MatScores. To ensure that our subsample did not differ significantly from the larger sample, we compared birth weights of animals from the two subsamples using t-tests. The means of each subset (mean_hist_ = 28.77 g; mean_exp_ = 29.46 g) did not differ (t(200) = 1.136, p = 0.260). Finally, our analyses of urinary androgens and PPNAS-M behaviours are exclusively derived from data collected from the animals born from January 2016–June 2016.

Our sample consisted of marmosets born to twin and triplet litters of known sex composition, and we compared monkeys born to mixed-sex (i.e., at least one full-term male and female) litters to monkeys from same-sex (i.e., all male or all female) litters (Table 1). Using this partitioning strategy, we could concomitantly investigate whether androgens from males or other, untested, types of sex-dependent interactions might be driving variation in infants’ developmental outcomes. All study subjects were housed as members of family groups (i.e., infants, parents, and older siblings). Marmosets’ enclosures measured approximately 182 × 152 × 91 cm and were equipped with wooden branches and nest boxes. The SNPRC maintains marmosets on a 12-hour light/dark schedule, with temperatures ranging from 24–30 °C. Adults’ diets consist of either ZuPreem Marmoset Diet (Premium Nutritional Products, Inc., Shawnee, KS) or Mazuri Callitrichid High Fiber Diet (Land O Lakes, Mazuri, Brentwood, MO), which was provided each morning. Marmosets also received daily supplements of fruit, vegetables, and yogurt each afternoon. *Ad libitum* water was available to all animals, and animals were monitored daily for general condition, appearance, and dietary intake/output.

### Urine Collection

To examine the levels of urinary androgens in neonates, we collected urine from newborns 24–36 hours after parturition. All births occurred at night, which is typical of marmoset parturitions^78^. We did not disturb any infants on the day of parturition (i.e., postnatal day (PD) 0). The following morning (PD 01, 7–8 AM), we removed infants from their family groups for collection of urine, morphometric measurements, and behavioural testing. We relocated newborns to a separate room and placed them on a heated surrogate (i.e., a stuffed animal) to prevent hypothermia. We also collected urine from infants on PD 15 and PD 30 (±2 days), following the same protocol for PD 01, to investigate whether gestation with brothers influenced the production of androgens over the period of infancy.

We collected urine from infants by using absorbent surgical eye spears (Beaver Visitec International Ltd Eye Spears, Fisher Scientific) to gently stimulate the anogenital region. This mimics alloparental care and induces urination and defecation in marmoset infants^79,80^. We centrifuged saturated eye spears in 1.5 ml microcentrifuge tubes for 15 minutes at 7,000 RPM, discarded the spears, then stored the samples at −20 °C until assays were performed at the Callitrichid Research Center at the University of Nebraska, Omaha.

### Androgen Assay

To determine the concentrations of urinary androgens in infants, we used an enzyme immunoassay (EIA) that previously has been validated for marmosets^81^ and described in detail by French *et al*.^36^. Briefly, for each microtiter plate, we included a standard curve (1000-7.8 pg androgens), two sets of quality-controlled pools (i.e., pooled marmoset urine) that represented high and low concentration quality controls, and experimental samples. These controls and experimental samples were assayed in duplicate to generate interassay coefficients of variation (CV). These CVs averaged to 4.53% and 16.01% for the high and low concentration pools, respectively. Furthermore, we refer to our results generally as “androgen” concentrations because the antibody in this assay is known to cross-react with androstenedione and dihydrotestosterone (other steroid cross-reactivity <2%)^35,82^.

While EIAs typically control for inter-individual variation in fluid intake and urinary output by concurrently assaying for creatinine, we lacked sufficient volumes of urine to assay for both androgen and creatinine. We therefore express resulting androgen concentrations as ng/ml urine, rather than μg/mg creatinine. Despite this limitation, we did attempt to control for variability in urinary output by sampling infants first thing in the morning. We therefore expect that the samples we collected represented infants’ first voids. This expectation is supported by known patterns of nighttime maternal care in Wied’s black tufted-ear marmoset (*Callithrix kuhlii*)^78^. That is, while marmoset mothers wake repeatedly to care for offspring, arousals usually occur during the late-night hours (20:00–23:59) rather than the early or late morning hours (0:00–7:00)^78^. As such, we assume that infants had likely nursed at approximately the same time the night preceding testing and consequently might have comparable urinary outputs during first voids. For some instances, infants produced urine samples following behavioural assays. These samples did not differ from samples produced before behavioural testing (t(55) = 0.921, p = 0.361), so we included these samples in subsequent analyses. Considering these limitations of our data, we cautiously present these results as representing valid differences in infants’ urinary androgens.

The clearance of androgens from peripheral circulation through urine is known to take approximately 36 hours in common marmosets, based upon labeled hormone studies^83^. Ziegler *et al*.^41^ also demonstrated that testosterone metabolites can be detected in callitrichine urine up to 48 hours following injection. Therefore, it is reasonable to assume that the urinary androgen concentration at ~36 hours after birth is a reflection of peripheral circulation at the time of parturition.

### Morphometrics

We used calipers to measure biparietal diameters, knee-heel lengths, thigh-knee lengths, and upper-arm lengths for each study subject on PD 01, 15 and 30. We used string to measure individuals’ abdominal circumferences and crown-rump lengths. We conducted all these morphometric measurements in triplicate to the nearest 0.01 cm. We also collected weights on PD 01, 15, and 30 to the nearest gram.

We collected data corresponding to infants’ 2^nd^ and 4^th^ phalanx lengths using two methodologies. First, we inspected photographs of infants’ hands and feet to determine digit ratios. We also determined infants’ 2^nd^ and 4^th^ phalanx lengths by measuring neonatal handprints. We then generated digital lengths using ImageJ^84,85^ and summed the lengths of the proximal, intermediate, and distal phalanxes to obtain the total lengths for each digit. We conducted all measures in triplicate. We then calculated the ratio of the lengths of the second to the fourth digits to obtain the 2D:4D ratio^86^. Because BMF and an undergraduate research assistant generated the 2D:4D ratios, we calculated intraclass correlation coefficients (ICC)^87,88^ and 95% confidence intervals of the 2D:4D ratios using R package “ICC”^89^ based on a mean-rating (k = 2) (ICC = 0.886 ± 0.079).

#### Growth

We weighed infants on PD 01, 15, and 30 (7–9 AM) to the nearest gram to determine perinatal weights and growth rates (grams/day) for each study subject.

### Infant Neurodevelopment and Behaviour

We conducted Marmoset Assessment Tests (“MatScore”)^38^ 24–36 hours after birth. This behavioural assay generates scores based on newborns’ responses to seven tasks that to gauge motor and sensory skills for newborn marmosets. For detailed description of the assessment protocol, refer to Supplementary Table 2. We also used the Primate Postnatal Neurobehavioural Assessment Scale for Marmosets (“PPNAS-M”)^39^ to assess neurodevelopment and behaviour in common marmosets on PD 15 and 30. This assay involves 41 noninvasive tests to measure orienting capability, motor skills, righting, strength, and temperament in marmoset infants. For detailed description of the assessment protocol, refer to Supplementary Table 3.

### Statistical Analyses

To assess whether the sex composition of litters impacts levels of urinary androgens in perinatal marmosets, we constructed generalized linear mixed models (GLMM) using the R package “lme4”^90^. We analyzed the data corresponding to PD 01 separately from the data for PD 15 and 30 because we cannot assume that the physiological mechanisms underlying androgen profiles on the first day of life are the same mechanisms underlying androgen profiles later in ontogeny^41^. In GLMMs for androgen concentrations on PD 01, we included sex a fixed effect to account for potential sexual dimorphism in androgen physiology. We also included litter identity as a random effect to account for variation among maternal androgen profiles during gestation. In GLMMs for androgen concentrations on PD 15 and PD 30, we expanded the model to include the sampling date as a fixed effect (i.e., 2 levels; PD 15 and PD 30) and nested infants’ identities within that of litters to account for non-independence of repeated measures.

We generated GLMMs to explore morphological differences (i.e., perinatal weight, biparietal diameter, knee-heel length, upper-arm length, thigh-length, abdominal circumference, crown-rump length, and 2D:4D ratios) in the infants. We tested for collinearity of all morphometric measurements using the R package “ppcor”^91^ and eliminated covariates that showed collinearity with other variables (p < 0.05). In resulting models, we included the day we collected measurements (PD 01, PD 15, or PD 30) as a fixed effect and infant identity, nested within that of litters, as a random effect. For our analysis of 2D:4D ratios, we included the method of measurement (i.e., photos or prints) as a fixed effect.

We constructed cumulative link mixed models (CLMMs) using the R package “ordinal”^92^ to investigate the links between litter sex composition and behaviour. We conducted two sets of analyses corresponding to the MatScore and PPNAS-M protocols. For models comparing same- versus mixed-sex litters in MatScore results, we also included sex as a predictor to examine any sexual dimorphism between male and female marmosets. In models investigating PPNAS-M scores, we also included the day of testing (i.e., PD 15 or 30) as a fixed effect and identity as a random effect to account for repeated measures of study subjects. All statistical analyses were conducted in R (R Core Team, 2016).

### Ethical Note

We conducted this study in accordance with the recommendations outlined in the National Research Council Guide for the Care and Use of Laboratory Animals (2011) and the American Society of Primatologists Guidelines for the Use of Animals in Research. The SNPRC is fully accredited by the Council on Accreditation of the Association for Assessment and Accreditation of Laboratory Animal Care (AAALAC International). The Institutional Animal Care and Use Committee at the Texas Biomedical Research Institute approved this experimental protocol.

## Supplementary information

Supplementary Information

## Data Availability

The datasets generated during and/or analyzed during the current study are not publicly available because all health and research data is under the purview of the SNPRC. However, datasets may be available from Suzette Tardif on reasonable request.

## References

[1] Birnie AK, Hendricks SE, Smith AS, Milam R, French JA (2012). Maternal gestational androgens are associated with decreased juvenile play in white-faced marmosets (*Callithrix geoffroyi*). Horm. Behav..

[2] Ventura T, Gomes MC, Pita A, Neto MT, Taylor A (2013). Digit ratio (2D:4D) in newborns: influences of prenatal testosterone and maternal environment. Early Hum. Dev..

[3] Berghänel A, Heistermann M, Schülke O, Ostner J (2016). Prenatal stress effects in a wild, long-lived primate: predictive adaptive responses in an unpredictable environment. Proc. R. Soc. Lond. B..

[4] Wallen, K. & Baum, M. J. Masculinization and defeminization in altricial and precocial mammals: comparative aspects of steroid hormone action. (eds Pfaff, D. W., Arnold, A. P., Etgen, A. M., Fahrbach, S. E. & Rubin, R. T.) *Hormones, Brain and Behaviour* (Vol. 4, pp. 385–423) (2002).

[5] Dufty AM, Clobert J, Møller AP (2002). Hormones, developmental plasticity and adaptation. Trends Ecol. Evol..

[6] Nettle D, Bateson M (2015). Adaptive developmental plasticity: what is it, how can we recognize it and when can it evolve?. Proc. R. Soc. Lond. B..

[7] Laplante DP, Brunet A, King S (2015). The effects of maternal stress and illness during pregnancy on infant temperament: Project Ice Storm. Pediatr. Res..

[8] Miranda A, Sousa N (2018). Maternal hormonal milieu influence on fetal brain development. Brain Behav..

[9] Sheriff MJ (2017). Integrating ecological and evolutionary context in the study of maternal stress. Integr. Comp. Biol..

[10] Huhtaniemi I (1994). Fetal testis-a very special endocrine organ. Eur. J. Endocrinol.

[11] Slob AK, Ooms MP, Vreeburg JTM (1980). Prenatal and early postnatal sex differences in plasma and gonadal testosterone and plasma luteinizing hormone in female and male rats. J. Endocrinol..

[12] Quinn A, Koopman P (2012). The molecular genetics of sex determination and sex reversal in mammals. Semin. Reprod. Med..

[13] Kaludjerovic, J. & Ward, W. E. The interplay between estrogen and fetal adrenal cortex. *J. Clin. Nutr. Med.* **2012**, 1–12 (2012). 10.1155/2012/837901 PMC3321452 22536492

[14] Albrecht ED, Pepe GJ (2010). Estrogen regulation of placental angiogenesis and fetal ovarian development during primate pregnancy. Int. J. Dev. Biol..

[15] Bakker J, Brock O (2010). Early estrogens in shaping reproductive networks: evidence for a potential organizational role of estradiol in female brain development. J. Neuroendocrinol..

[16] Mitchell RT (2008). Germ cell differentiation in the marmoset (*Callithrix jacchus*) during fetal and neonatal life closely parallels that in the human. Hum. Reprod..

[17] Even MD, Dhar MG, Saal vom FS (1992). Transport of steroids between fetuses via amniotic fluid in relation to the intrauterine position phenomenon in rats. J. Reprod. Fertil..

[18] Ryan BC, Vandenbergh JG (2002). Intrauterine position effects. Neurosci. Biobehav. Rev..

[19] Berenbaum SA, Bryk KLK, Beltz AM (2012). Early androgen effects on spatial and mechanical abilities: evidence from congenital adrenal hyperplasia. Behav. Neurosci..

[20] Puts DA, McDaniel MA, Jordan CL, Breedlove SM (2008). Spatial ability and prenatal androgens: meta-analyses of congenital adrenal hyperplasia and digit ratio (2D:4D) studies. Arch. Sex. Behav..

[21] Roof RL, Havens MD (1992). Testosterone improves maze performance and induces development of a male hippocampus in females. Brain Res..

[22] Jonasson Z (2005). Meta-analysis of sex differences in rodent models of learning and memory: a review of behavioural and biological data. Neurosci. Biobehav. Rev..

[23] Range F, Bugnyar T, Schlögl C, Kotrschal K (2006). Individual and sex differences in learning abilities of ravens. Behav. Proc..

[24] Voyer D, Postma A, Brake B, Imperato-McGinley J (2007). Gender differences in object location memory: a meta-analysis. Psycho. Bull. Rev..

[25] Abbott AD, Colman RJ, Tiefenthaler R, Dumesic DA, Abbott DH (2012). Early-to-mid gestation fetal testosterone increases right hand 2D:4D finger length ratio in polycystic ovary syndrome-like monkeys. PLoS ONE.

[26] Hackländer K, Arnold W (2011). Litter sex ratio affects lifetime reproductive success of free-living female Alpine marmots *Marmota marmota*. Mammal Rev..

[27] Monclús R, Holst, von D, Blumstein DT, Rödel HG (2014). Long-term effects of litter sex ratio on female reproduction in two iteroparous mammals. Funct. Ecol..

[28] Korsten P, Clutton-Brock T, Pilkington JG, Pemberton JM, Kruuk LEB (2009). Sexual conflict in twins: male co-twins reduce fitness of female Soay sheep. Biol. Lett..

[29] Drickamer LC, Arthur RD, Rosenthal TL (1997). Conception failure in swine: importance of the sex ratio of a female’s birth litter and tests of other factors. J. Anim. Sci..

[30] Bradley BJ (2016). Non‐human primates avoid the detrimental effects of prenatal androgen exposure in mixed‐sex litters: combined demographic, behavioural, and genetic analyses. Am. J. Primatol..

[31] French JA (2016). Gene changes may minimize masculinizing and defeminizing influences of exposure to male cotwins in female callitrichine primates. Biol. Sex. Differ..

[32] Rutherford JN, deMartelly VA, Layne Colon DG, Ross CN, Tardif SD (2014). Developmental origins of pregnancy loss in the adult female common marmoset monkey (*Callithrix jacchus*). PLoS One.

[33] Tardif SD, Jaquish CE (1997). Number of ovulations in the marmoset monkey (*Callithrix jacchus*): relation to body weight, age and repeatability. Am. J. Primatol.

[34] Tardif SD (2003). Reproduction in captive common marmosets (*Callithrix jacchus*). Comp. Med..

[35] Hauser B, Deschner T, Boesch C (2008). Development of a liquid chromatography–tandem mass spectrometry method for the determination of 23 endogenous steroids in small quantities of primate urine. Journal of Chromatography B.

[36] French JA, Smith AS, Birnie AK (2010). Maternal gestational androgen levels in female marmosets (*Callithrix geoffroyi*) vary across trimesters but do not vary with the sex ratio of litters. Gen. Comp. Endocrinol.

[37] Rey R, Lukas-Croisier C, Lasala C, Bedecarrás P (2003). AMH/MIS: what we know already about the gene, the protein and its regulation. Mol. Cell Endocrinol..

[38] Tardif SD, Layne DG, Cancino L, Smucny DA (2002). Neonatal behavioral scoring of common marmosets (*Callithrix jacchus*): relation to physical condition and survival. J. Med. Primatol..

[39] Braun K, Schultz-Darken N, Schneider M, Moore CF, Emborg ME (2015). Development of a novel postnatal neurobehavioural scale for evaluation of common marmoset monkeys. Am. J. Primatol..

[40] Weisz J, Ward IL (1980). Plasma testosterone and progesterone titers of pregnant rats, their male and female fetuses, and neonatal offspring. Endocrinol..

[41] Ziegler TE, Carlson AA, Ginther AJ, Snowdon CT (2000). Gonadal source of testosterone metabolites in urine of male cotton-top tamarin monkeys (*Saguinus oedipus*). Gen. Comp. Endocrinol..

[42] Keelan JA (2012). Androgen concentrations in umbilical cord blood and their association with maternal, fetal and obstetric factors. PLoS One.

[43] Abbott DH, Hearn JP (1978). Physical, hormonal and behavioural aspects of sexual development in the marmoset monkey. Callithrix jacchus. J. Reprod. Fertil..

[44] Ziegler TE, Prudom SL, Zahed SR (2009). Variations in male parenting behaviour and physiology in the common marmoset. Am. J. Hum. Biol..

[45] Addessi E, Chiarotti F, Visalberghi E, Anzenberger G (2007). Response to novel food and the role of social influences in common marmosets (*Callithrix jacchus*) and Goeldi’s monkeys (*Callimico goeldii*). Am. J. Primatol..

[46] Shiba Y (2014). Individual differences in behavioural and cardiovascular reactivity to emotive stimuli and their relationship to cognitive flexibility in a primate model of trait anxiety. Front. Behav. Neurosci..

[47] Koski SE, Burkart JM (2015). Common marmosets show social plasticity and group-level similarity in personality. Sci. Rep..

[48] Rutherford JN, Tardif SD (2009). Developmental plasticity of the microscopic placental architecture in relation to litter size variation in the common marmoset monkey (*Callithrix jacchus*). Placenta.

[49] Tardif SD, Layne DG, Smucny DA (2002). Can marmoset mothers count to three? Effect of litter size on mother-infant interactions. Ethol..

[50] Levine J (1982). Rapid regression of fetal adrenal zone and absence of adrenal reticular zone in the marmoset. Endocrinol..

[51] Pattison, J. C. *et al*. Male marmoset monkeys express an adrenal fetal zone at birth, but not a zona reticularis in adulthood. *Endocrinol.* **146**, 365–74 (2005). 10.1210/en.2004-0689 15459122

[52] Pattison JC, Abbott DH, Saltzman W, Conley AJ, Bird IM (2009). Plasticity of the zona reticularis in the adult marmoset adrenal cortex: voyages of discovery in the New World. J. Endocrinol..

[53] Leigh SR (1992). Patterns of variation in the ontogeny of primate body size dimorphism. J. Hum. Evol..

[54] Smith RJ, Leigh SR (1998). Sexual dimorphism in primate neonatal body mass. J. Hum. Evol..

[55] Mills DA, Windle CP, Baker HF, Ridley RM (2004). Analysis of infant carrying in large, well-established family groups of captive marmosets (*Callithrix jacchus*). Primates.

[56] Santos C, French JA, Otta E (1997). Infant carrying behaviour in callitrichid primates: *Callithrix* and *Leontopithecus*. Int. J. Primatol..

[57] Fite JE (2005). Opportunistic mothers: female marmosets (*Callithrix kuhlii*) reduce their investment in offspring when they have to, and when they can. J. Hum. Evol..

[58] Tardif, S. D., Ross, C. & Smucny, D. Building marmoset babies: trade-offs and cutting bait. (eds Clancy, K. B. H., Hinde, K. & Rutherford, J. N.) *Building Babies: Primate Development in Proximate and Ultimate**Perspectives* (pp. 169–183) New York, NY: Springer New York (2013).

[59] Schradin C, Anzenberger G (2001). Costs of infant carrying in common marmosets, *Callithrix jacchus*: an experimental analysis. Ani. Behav..

[60] Tardif SD (1994). Relative energetic cost of infant care in small-bodied neotropical primates and its relation to infant-care patterns. Int. J. Primatol..

[61] Ross CN, French JA, Orti G (2007). Germ-line chimerism and paternal care in marmosets (*Callithrix kuhlii*). Proc. R. Soc. Lond. B..

[62] Sweeney CG, Curran E, Westmoreland SV, Mansfield KG, Vallender E (2012). Quantitative molecular assessment of chimerism across tissues in marmosets and tamarins. BMC Genomics.

[63] Wedi E (2016). Detection of cross-sex chimerism in the common marmoset monkey (*Callithrix jacchus*) in interphase cells using fluorescence *in situ* hybridisation probes specific for the marmoset X and Y chromosomes. Reprod. Fertil. Dev..

[64] Digby, L. J., Ferrari, S. F. & Saltzman, W. Callitrichines: The role of competition in cooperatively breeding species. In C. J. Campbell, A. Fuentes, K. C. MacKinnon, S. K. Bearder & R. M. Stumpf (Eds), *Primates in Perspective* (pp. 91–107). Oxford (2011).

[65] Henry MD, Hankerson SJ, Siani JM, French JA, Dietz JM (2013). High rates of pregnancy loss by subordinates leads to high reproductive skew in wild golden lion tamarins (*Leontopithecus rosalia*). Horm. Behav..

[66] Saltzman W, Digby LJ, Abbott DH (2009). Reproductive skew in female common marmosets: what can proximate mechanisms tell us about ultimate causes?. Proc. R. Soc. Lond. B..

[67] Sutcliffe AG, Poole TB (1984). Intragroup agonistic behaviour in captive groups of the common marmoset *Callithrix jacchus jacchus*. Int. J. Primatol..

[68] Rothe H, Konig A, Radespiel U, Darms K, Siess M (1988). Occurrence and frequency of twin-fight in the common marmoset (*Callithrix jacchus*). Z. Saugetierkunde.

[69] Perier A, Lebrun R, Marivaux L (2016). Different level of intraspecific variation of the bony labyrinth morphology in slow- versus fast-moving primates. J. Mammal. Evol..

[70] Young JW, Shapiro LJ (2018). Developments in development: what have we learned from primate locomotor ontogeny?. Am. J. Phys. Anth..

[71] de Sousa, M. B. C., da Rocha Alburquerque, A. C. S., Yamamoto, M. E., Araujo, A. & de Fatima Arruda, M. Emigration as a reproductive strategy of the common marmoset. In *The Smallest Anthropoids: The Marmoset/Callimico Radiation* (pp. 167–181). Springer Science+Business Media (2009).

[72] French JA, Cavanaugh J, Mustoe AC, Carp SB, Womack SL (2017). Social monogamy in nonhuman primates: phylogeny, phenotype, and physiology. J. Sex. Res..

[73] Vatten LJ, Skjærven R (2004). Offspring sex and pregnancy outcome by length of gestation. Early Hum. Dev..

[74] Chen SJ, Vohr BR, Oh W (1993). Effects of birth order, gender, and intrauterine growth retardation on the outcome of very low birth weight in twins. J. Pediatr..

[75] Koenig A (1995). Group size, composition, and reproductive success in wild common marmosets (*Callithrix jacchus*). Am. J. Primatol..

[76] Bales KL, Dietz J, Baker A, Miller K, Tardif SD (2000). Effects of allocare-givers on fitness of infants and parents in callitrichid primates. Fol. Primatol..

[77] Burkart JM (2015). Opposite effects of male and female helpers on social tolerance and proactive prosociality in callitrichid family groups. Sci. Rep..

[78] Fite, J. E. *et al*. Nighttime wakefulness associated with infant rearing in *Callithrix kuhlii. Int. J. Primatol.* **24**, 1267–1280 (2003).

[79] Kaplan G, Rogers LJ (1999). Parental care in marmosets (*Callithrix jacchus jacchus*): development and effect of anogenital licking on exploration. J. Comp. Psych..

[80] Stevenson MF (1976). Birth and perinatal behaviour in family groups of the common marmoset (*Callithrix j. jacchus*) compared to other primates. J. Hum. Evol..

[81] Nunes S, Fite JE, French JA (2000). Variation in steroid hormones associated with infant care behaviour and experience in male marmosets (*Callithrix kuhlii*). Anim. Behav..

[82] Dloniak SM (2004). E. Non-invasive monitoring of fecal androgens in spotted hyenas (*Crocuta crocuta*). Gen. Comp. Endocrinol..

[83] Möhle U, Heistermann M, Palme R, Hodges JK (2002). Characterization of urinary and fecal metabolites of testosterone and their measurement for assessing gonadal endocrine function in male nonhuman primates. Gen. Comp. Endocrinol..

[84] Schneider CA, Rasband WS, Eliceiri KW (2012). NIH Image to ImageJ: 25 years of image analysis. Nat. Meth..

[85] Rueden CT (2017). ImageJ2: ImageJ for the next generation of scientific image data. BMC Bioinform..

[86] McIntyre MH (2006). The use of digit ratios as markers for perinatal androgen action. Reprod. Biol. Endocrinol..

[87] Shrout PE, Fleiss JL (1979). Intraclass correlations: uses in assessing rater reliability. Psych. Bull..

[88] Koo TK, Li MY (2016). A guideline of selecting and reporting intraclass correlation coefficients for reliability research. J. Chiropr. Med..

[89] Wolak ME, Fairbairn DJ, Paulsen YR (2012). Guidelines for estimating repeatability. Meth. Ecol. Evol..

[90] Bates D, Maechler M, Bolker B, Walker S (2015). Fitting linear mixed-effects models usinglme4. J. Stat. Soft..

[91] Kim, S. ppcor – Partial and semi-partial (part) correlation. R package version 1.1, https://CRAN.R-project.org/package=ppcor (2015).

[92] Christensen, R. H. B. ordinal – Regression models for ordinal data. R package version 2018.4-19, http://www.CRAN.R-project.org/package=ordinal/ (2018).

