# Supplementary material for: Sibling sex, but not androgens, shapes phenotypes in perinatal common marmosets (Callithrix jacchus)
Source: Sci Rep. 2019 Jan 31;9:1100. doi: 10.1038/s41598-018-37723-z (PMC6355804; doi:10.1038/s41598-018-37723-z)
Supplement: Supplementary file 1 — Supplementary Information [file 41598_2018_37723_MOESM1_ESM.pdf]

Sibling sex, but not androgens, shapes phenotypes in perinatal common marmosets (*Callithrix jacchus*)

Brett M. Frye*[1], Lisa G. Rapaport[1], Talia Melber[2,3], Michael W. Sears[1], Suzette D. Tardif[4]

SUPPLEMENTARY INFORMATION

Supplementary Figures

Supplementary Figure 1.

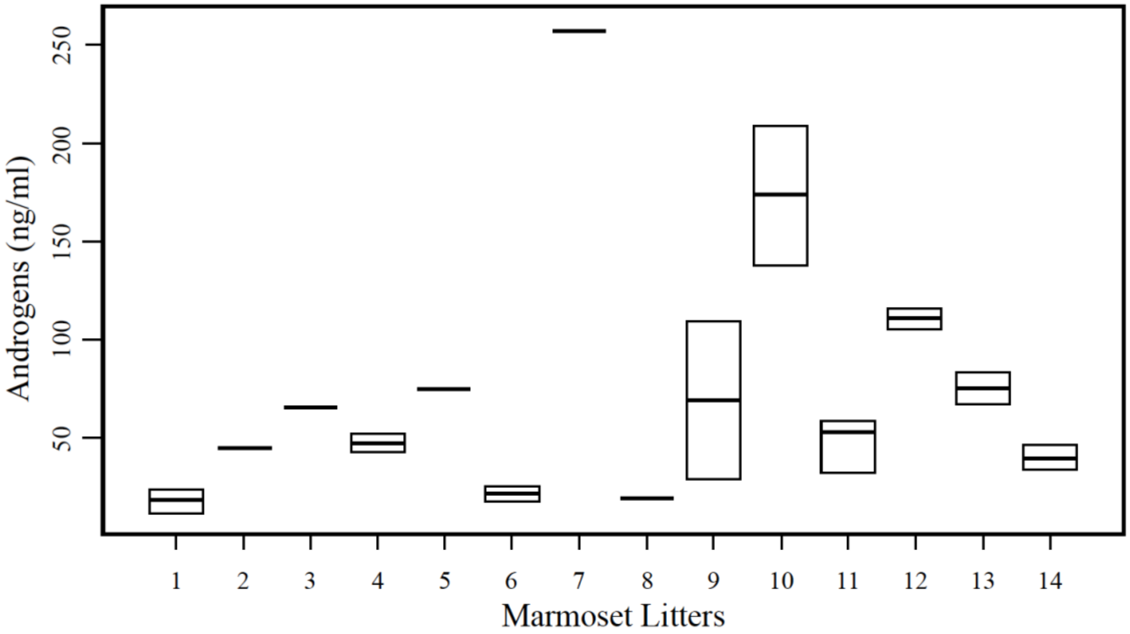

Supplementary Figure 1. Urinary androgens (ng/ml urine) in neonatal marmosets in same- and mixed-sex litters. Marmosets exhibited considerable variation in urinary androgen profiles among litters.

Supplementary Tables

Supplementary Table 1.

Task	Correlated Task(s)*
	MatScore
Clasping 2	Righting 1; Righting 2
Righting 2	Clasping 2; Righting 1
Rooting (left side)	Rooting (right side)
Auditory Orientation (left side)	Auditory Orientation (right side)
	PPNAS-M
Visual Following	Visual Orientation; Power
Duration of Looking	Visual Orientation; Attention; Power
Tactile Response	Rooting; Parachute Response; Range of Motion; Power
Range of Motion	Visual Orientation; Visual Following; Distractedness; Tactile Response; Response Speed and Intensity; Power
Power	Visual Orientation; Visual Following; Duration of Looking; Distractedness; Tactile Response; Response Intensity; Righting; Range of Motion; Stress to Limitation
Rooting	Distractedness; Rotation; Tactile Response; Galant’s Response; Crawling
Auditory Orientation	Visual Orientation; Visual Following; Distractedness; Tactile Response; Response Speed and Intensity; Righting; Range of

	Movement; Stress to Limitation
Crawling	Rooting; Coordination
Palmar Grasp	Inversion; Rotation; Plantar Grasp; Response Speed and Intensity
Response Speed	Auditory Orientation; Palmar Grasp; Labyrinthian Righting; Response Intensity; Range of Motion
Head Position (prone)	Startle Response; Inversion; Head Position (supine)
Head Position (supine)	Head Position (prone)

Supplementary Table 1. Behavioural tasks in MatScore and PPNAS-M procedures that showed evidence of multicollinearity to other tasks (Pearson Correlations $p < 0.05$). These tasks were eliminated from subsequent analyses examining the impact of litter type on behavioural outcomes in perinatal marmosets.

Supplementary Table 2.

Task	Score	Procedure	Scoring Criteria
Crawling	0 - 1	Infant placed on smooth surface	0 – Infant does not crawl<br>1 – Infant crawls
Clasping 1	0 - 2	Infant placed on dorsum; synthetic fur-covered cylinder brought into contact with infant’s	0 – Infant not able to hold on for entire time

		ventrum and cylinder raised 30 cm above test surface (1 minute)	1 – Infant clasps and holds; does not maintain ventral contact<br>2 – Infant clasps for entire time; maintains ventral contact
Righting 1	0 - 2	See ‘Clasping 1’ protocol	0 – Infant does not right itself<br>1 – Infant partially rights itself with at least half of body on bottom of cylinder<br>2 – Infant orients and climbs onto the side of the cylinder
Clasping 2	0 - 2	Infant placed upright on fur-covered cylinder, cylinder quickly rotated so infant’s dorsum almost touches test surface (30 minutes)	0 – Infant not able to hold on for entire time<br>1 – Infant clasps and holds; does not maintain ventral contact<br>2 – Infant clasps for entire time; maintains ventral contact

Righting 2	0 - 2	See ‘Clasping 2’ protocol	0 – Infant does not right itself<br>1 – Infant partially rights itself with at least half of body on bottom of cylinder<br>2 – Infant orients and climbs onto the side of the cylinder
Grasping 1	0 - 2	Infant placed on dorsum; experimenter’s gloved index finger placed within infant’s hands/feet and raised above the surface (1 minute)	0 – Infant does not grasp<br>1 – Infant grasps but does not hold for entire time<br>2 – Infant grasps for entire time
Grasping 2	0 - 2	Infant placed on dorsum; a wooden applicator stick is placed against the infant’s hand until infant holds. Infant is then pulled up and away from surface (30 sections)	0 – Infant does not grasp<br>1 – Infant grasps but does not hold for entire time<br>2 – Infant grasps for entire time
Vertical Orientation	0 - 2	Infant placed head down on a fur-covered board that is placed at a 45-degree angle from test surface	0 – Infant does not orient (i.e., remains still)

			1 – Partial orientation (i.e., infant turns approximately 90 degrees)<br>2 – Infant rotates 180 degrees within 60 seconds
Rooting	0 - 2	Plastic pipette tip is placed alongside, but not touching, each side of infant’s mouth	0 – No head turn toward object<br>1 – Infant turns head; no mouth contact<br>2 – Infant turns head and placing mouth on tip
Auditory Orientation	0 - 2	Infant held upright, and keys held behind and to the side of the infant’s head (out of visual range); keys shaken	0 – Infant does not turn head or move ears<br>1 – Infant partially responds-flicks ears<br>2 – Infant turns head toward direction of sound

Supplementary Table 2. Description of behavioural tests and scoring in the MatScore protocol (Tardif et al., 2002).

Supplementary Table 3.

Task	Score	Procedure	Scoring Criteria
Visual Orientation			

Visual Orientation	0 – 2	Orient infant away from tester, towards a blank wall. Bring brightly-coloured object approximately 30 cm away from the infant at a 45-degree angle in each periphery. Move object in four directions, removing the object between each direction: 1) Up-Left, 2) Down-Left, 3) Up-Right, and 4) Down-Right.	0 – Infant does not orient towards object<br>1 – Infant maintains brief orientation<br>2 – Infant maintains direct, prolonged orientation
Visual Following	0 – 2	Using previous task, assess the visual following of the bright toy in 1) vertical and 2) horizontal directions.	0 – Infant does not follow object<br>1 – Infant starts then stops following object<br>2 – Infant completely follows object
Reach and Grasping	0 – 2	Place the object/toy within arm's reach of the infant. Assess the ability of the monkey to reach for the toy during the visual orienting and following tests.	0 – No reach/grasp<br>1 – Swatting; no finger flexion<br>2 – Grasps with finger flexion
Duration of Looking	0 – 2	Assess the length of looking during the visual orienting and following tests.	0 – Infant does not look at the object<br>1 – Infant briefly glances at

			the (< 1 second)<br>2 – Prolonged looking at the object (> 1 second)
Distractibility	0 – 2	Rate distractedness during the visual orienting and following tests.	0 – No distraction<br>1 – Slight distraction<br>2 – Definite distraction
Attention	0 – 2	Rate attentiveness during the visual orienting and following tests.	0 – Infant is not attentive (< 25% of time)<br>1 – Infant maintains slight attention (25% time)<br>2 – Infant maintains definite attention (75% time)
Auditory and Spatial Orienting			
Auditory Startle	0 – 2	Use a set of keys; do not talk during test. Hold infant upright in one hand with infant's head facing away from you and then hold keys on one side of infant's head (approximately 100-degree angle from infant's nose, so just behind and off to one side of ear). Shake keys. Wait 30 seconds. Repeat on other side.	0 – Infant does not startle or whole-body jerk<br>1 – Infant slightly startles, eye jerk or ear flick<br>2 – Infant moderately startles, head jerk

Auditory Orientation	0 – 2	Assess infant’s ability to orient to “Auditory Startle” procedure.	0 – Infant does not orient<br>1 – Infant slightly orients, eye jerk or ear flick<br>2 – Infant turns head in direction of sound
Inversion	0 – 2	Hold the monkey in one hand and invert it approximately 30 cm from the testing surface. Hold monkey in inverted position for 30 seconds.	0 – Infant displays no aversion, no distress calls<br>1 – Infant displays slight aversion, distress calls 25% time<br>2 – Infant displays definite aversion, distress calls 75% time
Rotation	0 – 2	Place infant on surrogate or in clear tube. Spin infants for three complete rotations in each direction. Assess the eye/head turn in the direction of the spin.	0 – Infant does not turn head<br>1 – Infant weakly turns head, eyes<br>2 – Infant turns head fully in direction of spin
Motor Responses			
Tactile Response	0 – 2	Run a cotton tipped applicator along all four extremities against the	0 – Infant shows no response or exaggerated

		direction of the hair, distally to proximally; assess the response to the tactile stimulation.	response<br>1 – Infant shows barely discernable response<br>2 – Infant shows easily apparent response
Galant's Response	0 – 2	Run a cotton tipped applicator laterally along each side of the vertebral column with the direction of the hair. Assess any change in torso shape.	0 – No response or exaggerated response<br>1 – Slight curving of the spine<br>2 – Definite curving of the spine
Palmar Grasp	0 – 2	Induce hand grasp with the end of the cotton tipped applicator, sneak into palm while holding monkey.	0 – Infant grasps but then releases<br>1 – Infant grasps, digits stay closed<br>2 – Infant shows strong digit grasp without voluntary release
Plantar Grasp	0 – 2	Induce foot grasp with the end of the cotton tipped applicator, sneak into arch of foot while holding monkey.	0 – Infant grasps but then releases<br>1 – Infant grasps, digits stay closed<br>2 – Infant shows strong

			digit grasp without voluntary release
Rooting	0 – 2	Apply light tactile stimulus with the cotton-tipped applicator at the corner of the mouth but not directly in the mouth.	0 – No head turn toward the object<br>1 – Infant weakly turn of head towards stimulus, no mouth contact<br>2 – Infant promptly turns head and places mouth onto object
Parachute	0 – 2	While holding infant, invert the infant approximately 60 cm above the testing surface. With the monkey's arms free, assess the monkey's upper extremity limb extension during a headfirst descent towards a surface.	0 – No extension of arms<br>1 – Partial extension of arms<br>2 – Definite extension of arms/digits
Labyrinthian Righting	0 – 2	Holding the lower half of the infant (i.e., head and arms free), tilt the monkey's body 45 degrees sideways and assess realignment of the head in the left and right directions.	0 – Head stays in the plane of the body<br>1 – Head partially rights<br>2 – Head aligns with vertical plane
Vertical Orientation	0 – 2	Place infant on fur-covered board (27 x 35 cm) and place it against the wall	0 – Infant does not orient (i.e., remains still)

		or supported at approximately 45-degree angle from the test surface. Brush the fur “down.” Place the infant’s ventral surface onto the fur with its head toward the test surface. Release and begin time.	1 – Partial reorientation (i.e., infant turns 90 degrees)<br>2 – Infant fully orients with head now “up”
Response Speed	0 – 2	Assess the response speed of infant during the tests.	0 – 25% of responses are quick<br>1 – 75% of responses are quick<br>2 – All responses are quick
Response Intensity	0 – 2	Assess the response intensity of the tests, focusing on the quality of vocal responses.	0 – Vocalizations are mild in intensity<br>1 – Vocalizations are moderate/average<br>2 – Extremely loud/shrill vocalizations
Righting and Body Strength			
Body Righting	0 – 2	On a smooth, flat surface, place the monkey on its back and assess the time needed to turn from supine to prone.	0 – Infant does not turn over<br>1 – Infant turns over, but requires more than 2 s to

			do so<br>2 – Infant turns over instantaneously, < 2 s
Passive Range of Motion	0 – 2	Flex and extend each arm three times to assess the degree of resistance.	0 – Infant exhibits barely discernable resistance or exaggerated rigidity<br>1 – Infant exhibits mild resistance<br>2 – Infant exhibits moderate resistance
Active Power	0 – 2	In conjunction with the above test, assess the strength of the muscles when actively contracting.	0 – Cannot withstand slight resistance or exaggerated resistance<br>1 – Withstands mild resistance<br>2 – Withstands moderate resistance
Distress to Limitations	0 – 2	Hold infant completely (i.e., arms, legs, and tail) in hands. Restrict movement for 10 seconds and assess the degree of resistance/vocalizations.	0 – Continuous or complete lack of resistance or vocalizations<br>1 – Resistance or vocalizations 10% time<br>2 – Resistance or

			vocalizations approximately 25% time
Head Posture (Prone)	0 – 2	Hold monkey horizontally, prone (i.e., facing down). Assess the ability of the monkey to hold its head up.	0 – Flaccid head, hanging down<br>1 – Head lifted but not maintained<br>2 – Sustained lifting of the head with semiflexion
Head Posture (Supine)	0 – 2	Hold monkey horizontally, supine (i.e., facing up). Assess the ability of the monkey to hold its head up.	0 – Flaccid head, hanging down<br>1 – Head lifted but not maintained<br>2 – Sustained lifting of the head with semiflexion
Coordination	0 – 2	Assess the quality of the infant's movements during the motor and righting/strength tests.	0 – Clumsy movements<br>1 – Adequate movements<br>2 – Agile movements
Crawling	0 – 2	Place infant onto a smooth surface; release infant and start stopwatch.	0 – Infant does not crawl<br>1 – Infant crawls in uncoordinated fashion<br>2 – Infant crawls in coordinated fashion

Tremulousness	0 – 2	Assess the shakiness of the monkey during the tests.	0 – 3–4 events<br>1 – 1–2 events<br>2 – No tremulousness

Supplementary Table 3. Description of behavioural tests and scoring in the Primate Postnatal Neurobehavioural Assessment Scale for Marmosets (PPNAS-M) protocol (Braun et al., 2015).

Supplementary Table 4.

Response Variable	Fixed Effect	Estimate	SE	*t*	*P*
Bi-parietal diameter					
	Intercept	1.842	0.034	54.242	<0.001
	Litter Type	0.033	0.033	0.976	0.336
	Sex	0.008	0.018	0.436	0.664
	Litter Size	0.058	0.050	1.162	0.256
	Age*	-	-	-	-
	Interaction: Litter Type × Sex	0.010	0.065	0.157	0.876
Thigh Length					
	Intercept	2.269	0.052	43.272	<0.001
	Litter Type	0.030	0.050	0.606	0.549
	Sex	0.034	0.021	1.568	0.122
	Litter Size	-0.085	0.058	-1.481	0.152

	Age	0.807	0.023	35.515	<0.001
	Interaction: Litter Type × Sex	-0.151	0.097	-1.558	0.128
Upper Arm Length					
	Intercept	2.135	0.057	37.333	<0.001
	Litter Type	0.054	0.077	0.698	0.496
	Sex	-0.045	0.039	-1.158	0.255
	Litter Size	-0.036	0.065	-0.542	0.598
	Age*	-	-	-	-
	Interaction: Litter Type × Sex*	-	-	-	-
Abdominal Circumference					
	Intercept	5.561	0.218	25.530	<0.001
	Litter Type	0.166	0.304	0.548	0.592
	Sex	-0.035	0.139	-0.251	0.804
	Litter Size	0.036	0.256	0.140	0.891
	Age*	-	-	-	-
	Interaction: Litter Type × Sex*	-	-	-	-
Crown-rump Length (String)					
	Intercept	8.571	0.156	54.889	<0.001
	Litter Type	0.205	0.225	0.914	0.374
	Sex	-0.059	0.130	-0.456	0.651

Litter Size	-0.121	0.180	-0.674	0.514
Age*	-	-	-	-
Interaction: Litter Type × Sex*	-	-	-	-

Insufficient data for analysis

Supplementary Table 4. Generalized linear mixed models indicating relationship between and individual's litter type (i.e., same- versus mixed-sex), sex, litter size (i.e., twin or triplet), and age (i.e., postnatal day 01, 15, or 30).
